# Traditional Chinese Medicine Approach to Renal Cell Carcinoma: Jing-Si Herbal Tea Triggers Apoptosis and Ferroptosis in HK-2 Cells

**DOI:** 10.5812/ijpr-166286

**Published:** 2025-11-15

**Authors:** Yao-Chou Tsai, Chung-Che Tsai, Jin‑Yin Chang, Hsu-Hung Chang, Chan-Yen Kuo

**Affiliations:** 1 Division of Urology, Department of Surgery, Taipei Tzuchi Hospital, The Buddhist Tzu Chi Medical Foundation, New Taipei City, Taiwan; 2 Department of Research, Taipei Tzu Chi Hospital, The Buddhist Tzu Chi Medical Foundation, New Taipei City, Taiwan; 3 Department of Medical Research, Cathay General Hospital, Taipei City, Taiwan; 4 Division of Nephrology, Department of Internal Medicine, Sijhih Cathay General Hospital, New Taipei City, Taiwan; 5 Institute of Oral Medicine and Materials, College of Medicine, Tzu Chi University, Hualien, Taiwan

**Keywords:** Jing-Si Herbal Tea, Renal Cell Carcinoma, Apoptosis, Ferroptosis, GPX4, SLC7A11

## Abstract

**Background:**

Renal cell carcinoma (RCC) is the most common malignant tumor of the kidney, with clear cell renal cell carcinoma (ccRCC) representing its predominant subtype. Despite significant advances in targeted and immune therapies, treatment resistance and recurrence continue to pose major challenges, underscoring the urgent need for novel therapeutic strategies. Jing-Si Herbal Tea (JSHT), a traditional Chinese medicine (TCM) formulation, has demonstrated anti-tumor, anti-inflammatory, and antioxidant properties; however, its mechanisms of action in kidney disease and cancer remain poorly understood.

**Methods:**

Human proximal tubule epithelial (HK-2) cells were treated with various concentrations of JSHT (0%, 4%, and 8%) for 24 and 48 hours. Cell viability was assessed using the WST-1 assay. The expression levels of cleaved-caspase 3, glutathione peroxidase 4 (GPX4), and solute carrier family 7 member 11 (SLC7A11) were analyzed by western blotting and quantified relative to β-actin.

**Results:**

The JSHT significantly reduced HK-2 cell viability in a time- and concentration-dependent manner. Treatment with JSHT resulted in upregulation of cleaved-caspase 3, indicating activation of apoptosis, while concurrently downregulating GPX4 and SLC7A11, which are key regulators of ferroptosis resistance. These findings demonstrate that JSHT induces two forms of regulated cell death — ferroptosis and apoptosis — in renal epithelial cells.

**Conclusions:**

The JSHT exhibits antiproliferative effects in HK-2 cells by promoting apoptosis and sensitizing cells to ferroptosis through suppression of GPX4 and SLC7A11. These results provide preliminary mechanistic insights into the effects of JSHT and indicate its potential relevance for future research in RCC. However, validation in RCC-specific models and in vivo systems is necessary. A notable limitation of this study is the use of a non-cancerous renal cell line; thus, the in vitro findings may not fully recapitulate tumor biology. Future studies employing RCC cell lines, patient-derived tumor models, and in vivo validation are warranted to elucidate the therapeutic potential of JSHT as a complementary approach for RCC management.

## 1. Background

Kidney cancer, a common malignancy of the urinary system, primarily originates from renal epithelial cells, with renal cell carcinoma (RCC) accounting for approximately 90% of all kidney cancers (1). Clear cell renal cell carcinoma (ccRCC) is the most prevalent subtype and is characterized by mutations in the von Hippel-Lindau gene, which lead to dysregulation of hypoxia-inducible factors and promote tumor growth and angiogenesis (2). The clinical management of kidney cancer remains challenging due to late-stage diagnosis, resistance to conventional treatments, and the inherent heterogeneity of tumor biology (3). Current therapeutic strategies include surgical resection, targeted therapies — such as vascular endothelial growth factor (VEGF) inhibitors and tyrosine kinase inhibitors — and immunotherapies, including immune checkpoint inhibitors (4). However, these treatments are often associated with adverse effects, limited efficacy in advanced disease, and high recurrence rates, emphasizing the urgent need for novel therapeutic interventions (5).

While surgery, immunotherapy, and targeted therapy remain the mainstays of management, traditional Chinese medicine (TCM) and alternative therapies are increasingly being considered as complementary approaches to improve patient outcomes (6). Jing-Si Herbal Tea (JSHT), a formulation rooted in TCM, has attracted attention for its potential therapeutic properties, including anti-inflammatory, antioxidant, and anti-tumor effects (7). The JSHT is composed of several herbal ingredients, such as *Camellia sinensis* (green tea), *Scutellaria baicalensis* (Chinese skullcap), and *Astragalus membranaceus* (huangqi), and is designed to harmonize the body's internal balance and enhance immune function (8-11). Previous studies have indicated that JSHT exerts cytotoxic effects on cancer cells, modulates the production of inflammatory cytokines, and reduces oxidative stress, making it a promising candidate for adjuvant cancer therapy (7, 12). In the context of kidney cancer, the bioactive compounds in JSHT may target key pathways involved in tumor progression, such as angiogenesis, apoptosis, and immune evasion. Moreover, this integrative approach aligns with the growing interest in complementary and alternative medicine for the management of cancer-related symptoms and the enhancement of quality of life.

Ferroptosis is a regulated form of non-apoptotic cell death characterized by iron-dependent lipid peroxidation (13). Unlike apoptosis, ferroptosis is initiated by the accumulation of lipid reactive oxygen species (ROS) and dysfunction of antioxidant defenses, particularly glutathione peroxidase 4 (GPX4) (14). The solute carrier family 7 member 11 (SLC7A11), which functions as a cystine/glutamate antiporter and an essential component of system Xc^-^, is implicated in carcinogenesis through the regulation of ferroptosis (15). The SLC7A11 maintains cellular redox homeostasis and supports tumor survival under oxidative stress by modulating intracellular cystine levels and extracellular glutamate release (16). Its overexpression is frequently observed in a variety of cancers and is associated with poor prognosis, tumor progression, and therapeutic resistance (17). The unique mechanisms underlying ferroptosis thus make it a promising therapeutic target in cancer, including kidney cancer, where conventional treatments often encounter resistance and relapse (18).

## 2. Objectives

Although JSHT has been investigated in various inflammatory and metabolic disease models, its role in the pathophysiology of kidney disease remains largely uncharacterized. Therefore, this study focuses on the in vitro mechanistic evaluation of JSHT, rather than its clinical application, to provide initial insights into its cellular effects.

## 3. Methods

### 3.1. Cell Line

The human proximal tubule epithelial (HK-2) cell line was obtained from the Bioresource Collection and Research Center (Taiwan). Cells were cultured in T-75 flasks (Corning Inc., USA) using Dulbecco’s Modified Eagle Medium (DMEM)/Ham’s F12 medium (Gibco, USA) supplemented with L-glutamine (2 mM), sodium pyruvate (1 mM), D-glucose (25 mM), penicillin-streptomycin (50 U/mL; Sigma, USA), and 10% heat-inactivated fetal bovine serum (FBS). Cultures were maintained at 37°C in a humidified atmosphere containing 95% air and 5% CO_2_, with medium changes every two days. Cells were passaged at 60 - 70% confluence using 0.25% trypsin-EDTA and utilized for experiments between passages 3 and 8. Routine mycoplasma testing was performed to confirm the absence of contamination.

### 3.2. Reagents

The JSHT (Catalog No.: #4711393151427), a novel TCM formula, was developed by the Buddhist Tzu Chi Medical Foundation, Taiwan. This standardized product was vacuum-sealed and comprised a carefully formulated blend of the following herbal ingredients: *Perilla frutescens* (Perillae Folium, Zi Su Ye, PF; 7.09%), *Houttuynia cordata* Thunb. (Houttuyniae Herba, Yu Xing Cao, HC; 14.18% by weight), *Anisomeles indica* (Yu Zhen Cao, AI; 21.28%), *Glycyrrhiza glabra* (Glycyrrhizae Radix et Rhizoma, Gan Cao, GG; 7.09%), *Ophiopogon japonicus* (Ophiopogonis Radix, Mai Men Dong, OJ; 14.18%), *Chrysanthemum morifolium* (Chrysanthemi Flos, Ju Hua, CM; 0.71%), *Platycodon grandiflorus* (Platycodonis Radix, Jie Geng, PG; 14.18%), and *Artemisia argyi* (Artemisiae Argyi Folium, Ai Ye, AA; 21.28%) (19). An aqueous extraction method was employed, followed by filtration and concentration to prepare JSHT. The final product was a standardized, vacuum-sealed dose containing 225 mL of concentrate. For cell culture experiments, the JSHT concentrate was diluted to achieve final concentrations of 4% and 8%. To exclude any solvent effects, a vehicle control group received medium containing 0.5% FBS without JSHT (0% JSHT). All treatments were performed in triplicate (n = 3 biological replicates).

### 3.3. Cell Viability Analysis

Cell viability was assessed using the WST-1 assay as previously described (20). Briefly, cells were plated at 5 × 10^4^ cells/mL per well in 24-well plates and cultured in phenol red-free DMEM supplemented with 0.5% heat-inactivated FBS for 24 hours. Cells were then treated with JSHT at 0%, 4%, and 8% for 24 and 48 hours. Following treatment, WST-1 reagent was added and cells were incubated for an additional 2 hours at 37°C. Absorbance at 450 nm was measured using a microplate reader (Thermo Fisher Scientific, USA).

### 3.4. Western Blot Analysis

Protein expression levels of cleaved caspase-3, GPX4, and SLC7A11 were evaluated by western blotting. The HK-2 cells were seeded in 6-well plates (2 × 10^5^ cells/well) and treated with JSHT as described above. Total protein was extracted using RIPA lysis buffer (Thermo Fisher Scientific, USA) containing protease and phosphatase inhibitors. Protein concentration was determined by BCA assay (Pierce, USA). Equal amounts of protein (30 µg per sample) were resolved using 10 - 12% SDS-PAGE and transferred to PVDF membranes (Millipore, USA). Membranes were blocked with 5% non-fat milk in TBST for 1 hour and incubated overnight at 4°C with primary antibodies: anti-cleaved caspase-3 (#9661, 1:1000; Cell Signaling Technology, USA), anti-GPX4 (A13309, 1:1000; ABclonal, USA), anti-SLC7A11 (A13685, 1:1000; ABclonal, USA), and anti-β-actin (AC038, 1:5000; ABclonal, USA). After washing, membranes were incubated with HRP-conjugated secondary antibodies (1:5000; Jackson ImmunoResearch, USA) for 1 hour at room temperature. Protein bands were visualized using ECL substrate (Bio-Rad, USA) and quantified with ImageJ software (NIH, USA). β-actin was used as the loading control.

### 3.5. Statistical Analyses

Data were analyzed using one- or two-way analysis of variance, as appropriate for the experimental design. When significant differences were detected, the Bonferroni post hoc test was employed for multiple comparisons using GraphPad Prism 9.0 (GraphPad Software, USA). Statistical significance was defined as P < 0.05 and P < 0.01. A P-value less than 0.05 was considered significant.

## 4. Results

### 4.1. Jing-Si Herbal Tea Reduces Cell Viability

**Figure 1.**
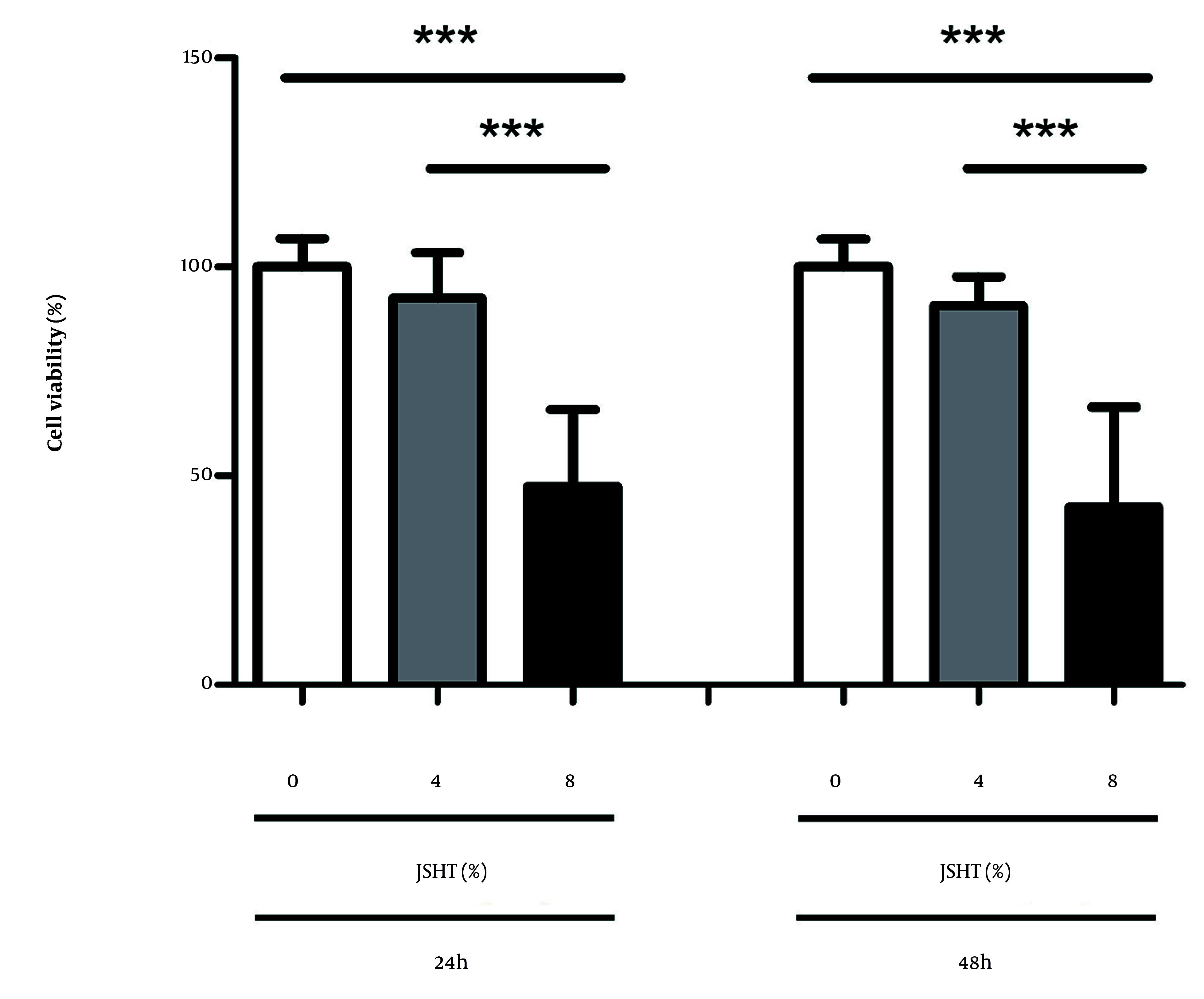
Effect of Jing-Si Herbal Tea (JSHT) on cell viability in human proximal tubule epithelial (HK-2) cells. The HK-2 cells were treated with various concentrations of JSHT (0%, 4%, and 8%) for 24 and 48 hours; the WST-1 assay assessed cell viability. The JSHT resulted in a significant, dose-dependent decrease in cell viability at both time points [values are expressed as the mean ± SD of three independent experiments; *** P < 0.001 vs. the controls (0%)].

The WST-1 assay demonstrated that JSHT significantly reduced the viability of HK-2 cells in a dose- and time-dependent manner (Figure 1). The JSHT treatment produced a significant, dose-dependent reduction in cell viability at both assessed time points. After 24 hours, exposure to 4% JSHT led to a modest decrease in cell viability compared with the untreated control group (0% JSHT), whereas 8% JSHT resulted in a pronounced reduction (P < 0.001). A similar pattern was observed at 48 hours, where 8% JSHT induced a greater decrease in viability than at 24 hours, indicating a time-dependent cytotoxic effect. Collectively, these results demonstrate that JSHT exerts significant inhibitory effects on HK-2 cell viability in vitro, in both a dose- and time-dependent manner (Figure 1). 

### 4.2. Jing-Si Herbal Tea Upregulates Cleaved Caspase-3 and Downregulates Glutathione Peroxidase 4/Solute Carrier Family 7 Member 11

**Figure 2.**
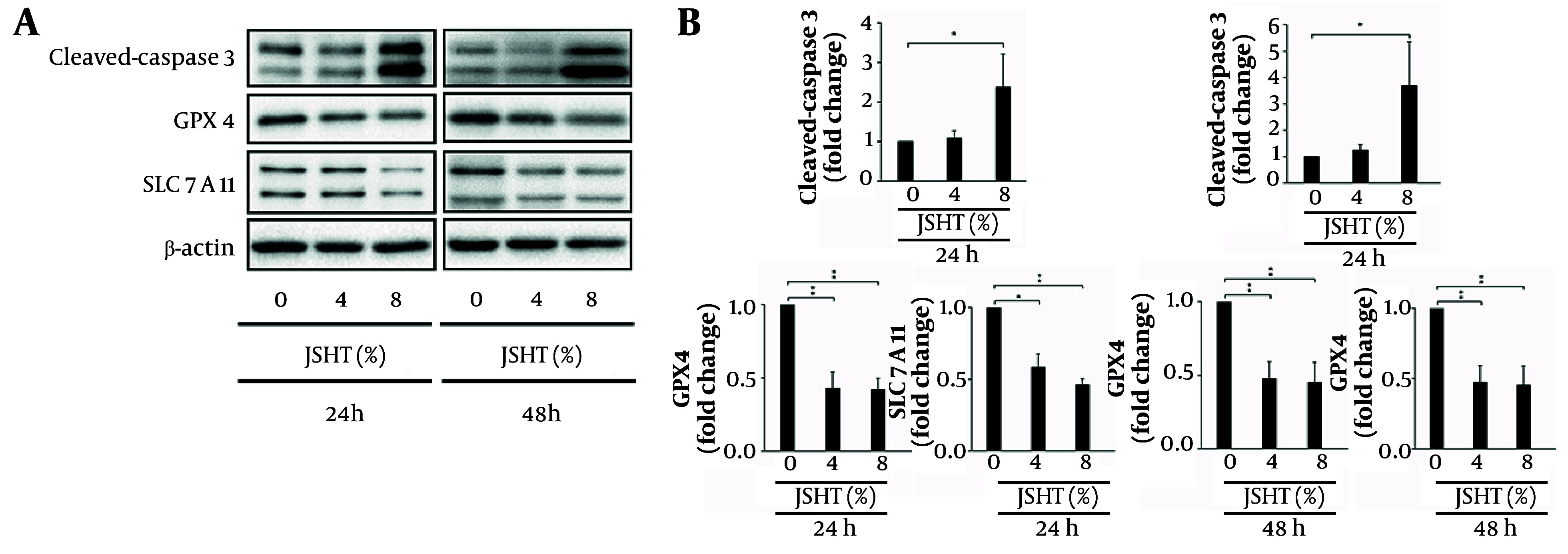
Effects of Jing-Si Herbal Tea (JSHT) on apoptosis- and ferroptosis-related proteins in human proximal tubule epithelial (HK-2) cells; A, western blot analysis of cleaved caspase-3, glutathione peroxidase 4 (GPX4), and solute carrier family 7 member 11 (SLC7A11) expression following treatment with JSHT (0%, 4%, and 8%) for 24 and 48 hours, β-actin was used as a loading control; B, quantification of protein expression levels, normalized to β-actin, is presented as fold change compared to untreated controls; JSHT significantly upregulated cleaved caspase-3 and downregulated GPX4 and SLC7A11 in a concentration-dependent manner (values represent mean ± SD of three independent experiments; * P < 0.05 and ** P < 0.01).

Caspases are crucial mediators of apoptosis (programmed cell death). Among them, caspase-3 is a key executioner, frequently activated to catalyze the cleavage of several important cellular proteins (21). The GPX4 acts as a major inhibitor of ferroptosis, playing a pivotal role in preventing this form of cell death (22). Conversely, SLC7A11, as an essential component of redox homeostasis, maintains cellular glutathione levels to counteract oxidative stress and inhibit ferroptosis (23). Western blot analysis revealed that JSHT significantly increased cleaved caspase-3 expression while decreasing GPX4 and SLC7A11 expression levels (Figure 2). 

After 24 hours of JSHT treatment, cleaved caspase-3 expression increased by 1.09-fold ± 0.18 (4% JSHT) and 2.37-fold ± 0.84 (8% JSHT) relative to control (1.00-fold). In contrast, GPX4 expression declined to 0.43 ± 0.11 (4% JSHT) and 0.42 ± 0.08 (8% JSHT), while SLC7A11 levels were reduced to 0.58 ± 0.09 (4% JSHT) and 0.46 ± 0.04 (8% JSHT), respectively. After 48 hours, these effects were further amplified: cleaved caspase-3 reached 1.24-fold ± 0.21 (4% JSHT) and 3.68-fold ± 1.66 (8% JSHT), whereas GPX4 was suppressed to 0.48 ± 0.12 (4% JSHT) and 0.45 ± 0.14 (8% JSHT), and SLC7A11 was reduced to 0.59 ± 0.01 (4% JSHT) and 0.56 ± 0.03 (8% JSHT), respectively. These findings indicate that JSHT exposure elevates cleaved caspase-3 expression while concurrently decreasing GPX4 and SLC7A11 levels in HK-2 cells (Figure 2). 

## 5. Discussion

The JSHT, a TCM formulation, exerts measurable molecular effects on HK-2 cells by modulating both apoptosis- and ferroptosis-associated markers. These results represent early mechanistic observations and provide a foundation for future investigation, rather than definitive therapeutic conclusions. Specifically, JSHT induced a significant, time- and dose-dependent reduction in cell viability, upregulated cleaved caspase-3, and downregulated the ferroptosis regulators GPX4 and SLC7A11. These findings highlight the dual potential of JSHT in activating programmed cell death and promoting ferroptotic vulnerability, thus offering mechanistic insights into its possible application as an adjunctive therapeutic strategy for RCC. However, these observations are preliminary and based solely on molecular indicators rather than direct functional assays.

### 5.1. Jing-Si Herbal Tea and Ferroptosis Modulation

The observed suppression of GPX4 and SLC7A11 expression underscores the ability of JSHT to promote ferroptosis. The GPX4 is a central inhibitor of ferroptosis, and its suppression leads to the accumulation of lipid peroxides, thereby facilitating ferroptotic cell death (22). Similarly, SLC7A11 is a critical component of the system Xc-cystine/glutamate antiporter, which maintains intracellular glutathione levels and protects cells against oxidative stress (24). Downregulation of both proteins implies that JSHT sensitizes renal epithelial cells to oxidative damage, shifting the balance toward ferroptosis. This is consistent with accumulating evidence that induction of ferroptosis may be an effective strategy for overcoming resistance in RCC — particularly ccRCC — where conventional therapies often fail due to metabolic adaptations and redox plasticity (25).

### 5.2. Crosstalk Between Apoptosis and Ferroptosis

In addition to promoting ferroptosis, JSHT also induced apoptosis, as evidenced by increased levels of cleaved caspase-3. While apoptosis and ferroptosis are distinct forms of regulated cell death, recent studies have demonstrated that agents capable of triggering both pathways may exert synergistic anti-tumor effects (26). The ability of JSHT to activate caspase-3-mediated apoptosis while concurrently suppressing ferroptosis regulators suggests that it may effectively circumvent tumor cell survival mechanisms, which often rely on resistance to a single mode of cell death. This dual regulatory action is particularly significant in RCC, where resistance to apoptosis is a hallmark of advanced disease progression.

### 5.3. Implications of Traditional Chinese Medicine in Oncology

The integrative activity of JSHT in targeting multiple pathways is consistent with the principles of TCM, which emphasize restoring systemic balance through multicomponent, multitarget interventions (27). The herbal constituents of JSHT — including *A. argyi*, *A. indica*, and *H. cordata* — are recognized for their anti-inflammatory, antioxidant, and immunomodulatory effects (7). Collectively, these compounds may create a synergistic network that not only induces tumor cell death but also modulates the tumor microenvironment, potentially enhancing the efficacy of existing therapeutic regimens. This approach aligns with the increasing interest in TCM-based formulations as complementary treatments to improve quality of life and minimize side effects in cancer patients.

### 5.4. Limitations and Recommendations

While this study provides important mechanistic insights, several limitations should be considered. Firstly, the experiments were limited to HK-2 cells, which, although widely used, do not fully recapitulate the complexity of ccRCC tumor cells and their microenvironment. Further validation using RCC cell lines and in vivo models is necessary to establish the relevance of these results. Secondly, the precise molecular mediators and signaling pathways downstream of GPX4 and SLC7A11 suppression remain unclear. Advanced omics approaches and genetic manipulation studies could clarify the mechanistic framework by which JSHT induces ferroptosis and apoptosis. Additionally, optimization studies, pharmacokinetics, and toxicity assessments are essential for evaluating the translational potential of JSHT in clinical settings.

Another limitation is that, although cleaved caspase-3, GPX4, and SLC7A11 are well-established markers of apoptosis and ferroptosis, this study did not include functional validation assays such as Annexin V/PI flow cytometry, lipid ROS quantification, or ferroptosis rescue experiments using ferrostatin-1 or liproxstatin-1. Due to constraints in funding and laboratory resources during the study period, these additional mechanistic experiments could not be performed. Therefore, the conclusions regarding the cell death pathways are based on molecular evidence and should be interpreted as preliminary. Future studies will incorporate ferroptosis- and apoptosis-specific functional assays to further validate the mechanistic effects of JSHT in renal carcinoma models.

### 5.5. Conclusions

**Figure 3.**
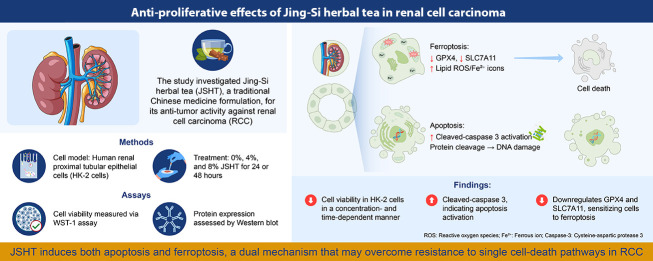
Graphical summary illustrating the anti-proliferative effects of Jing-Si Herbal Tea (JSHT) in renal cell carcinoma (RCC); human renal proximal tubular epithelial (HK-2) cells were treated with 0%, 4%, and 8% JSHT for 24 or 48 hours. Cell viability was assessed using the WST-1 assay, and protein expression was evaluated via Western blot. The JSHT treatment reduced cell viability in a time- and dose-dependent manner, induced apoptosis as evidenced by cleaved caspase-3 activation and DNA damage, and promoted ferroptosis through downregulation of glutathione peroxidase 4 (GPX4) and solute carrier family 7 member 11 (SLC7A11), accompanied by increased lipid reactive oxygen species (ROS) and Fe^2+^ accumulation. This dual induction of apoptosis and ferroptosis suggests that JSHT may overcome resistance associated with single-cell death pathways in RCC.

In summary, the findings indicate that JSHT exerts antiproliferative effects on renal epithelial cells by simultaneously promoting apoptosis and ferroptosis through caspase-3 activation and suppression of GPX4 and SLC7A11. This dual regulation demonstrates that JSHT modulates multiple cell death pathways at the molecular level. While these preliminary results support further investigation of JSHT in RCC-relevant models, therapeutic implications require additional validation (Figure 3). Ongoing and future studies using tumor-specific models and detailed mechanistic analyses will be essential to advance JSHT toward potential clinical application.

## Data Availability

All data generated or analyzed during this study are included in this published article. No additional datasets were generated or analyzed.

## References

[1] Hsieh JJ, Purdue MP, Signoretti S, Swanton C, Albiges L, Schmidinger M (2017). Renal cell carcinoma.. Nat Rev Dis Primers..

[2] Schodel J, Grampp S, Maher ER, Moch H, Ratcliffe PJ, Russo P (2016). Hypoxia, Hypoxia-inducible Transcription Factors, and Renal Cancer.. Eur Urol..

[3] Weiss RH, Lin PY (2006). Kidney cancer: identification of novel targets for therapy.. Kidney Int..

[4] Jonasch E, Atkins MB, Chowdhury S, Mainwaring P (2022). Combination of Anti-Angiogenics and Checkpoint Inhibitors for Renal Cell Carcinoma: Is the Whole Greater Than the Sum of Its Parts?. Cancers..

[5] Yang J, Wang K, Yang Z (2023). Treatment strategies for clear cell renal cell carcinoma: Past, present and future.. Front Oncol..

[6] Zheng J, Xu W, Liu W, Tang H, Lu J, Yu K (2021). Traditional Chinese medicine Bu-Shen-Jian-Pi-Fang attenuates glycolysis and immune escape in clear cell renal cell carcinoma: results based on network pharmacology.. Biosci Rep..

[7] Ho TJ, Ahmed T, Shibu MA, Lin YJ, Shih CY, Lin PY (2024). A prospective review of the health-promoting potential of Jing Si Herbal Tea.. Tzu Chi Med J..

[8] Prasanth MI, Sivamaruthi BS, Chaiyasut C, Tencomnao T (2019). A Review of the Role of Green Tea (Camellia sinensis) in Antiphotoaging, Stress Resistance, Neuroprotection, and Autophagy.. Nutrients..

[9] Yin B, Li W, Qin H, Yun J, Sun X (2021). The Use of Chinese Skullcap (Scutellaria baicalensis) and Its Extracts for Sustainable Animal Production.. Animals..

[10] Liang XL, Ji MM, Chen L, Liao Y, Kong XQ, Xu XQ (2021). Traditional Chinese herbal medicine Astragalus Radix and its effects on intestinal absorption of aconite alkaloids in rats.. Chin Herb Med..

[11] Wang CH, Yang JS, Chen CJ, Su SH, Yu HY, Juan YN (2024). Protective effects of Jing-Si-herbal-tea in inflammatory cytokines-induced cell injury on normal human lung fibroblast via multiomic platform analysis.. Tzu Chi Med J..

[12] Kao SW, Chang YC, Lin FH, Huang TL, Chen TS, Lin SZ (2024). Jing-Si Herbal Tea Suppresses H(2)O(2) -Instigated Inflammation and Apoptosis by Inhibiting Bax and Mitochondrial Cytochrome C Release in HIG-82 Synoviocytes.. Environ Toxicol..

[13] Pu F, Chen F, Zhang Z, Shi D, Zhong B, Lv X (2022). Ferroptosis as a novel form of regulated cell death: Implications in the pathogenesis, oncometabolism and treatment of human cancer.. Genes Dis..

[14] Kuang F, Liu J, Tang D, Kang R (2020). Oxidative Damage and Antioxidant Defense in Ferroptosis.. Front Cell Dev Biol..

[15] Yan Y, Teng H, Hang Q, Kondiparthi L, Lei G, Horbath A (2023). SLC7A11 expression level dictates differential responses to oxidative stress in cancer cells.. Nat Commun..

[16] Jyotsana N, Ta KT, DelGiorno KE (2022). The Role of Cystine/Glutamate Antiporter SLC7A11/xCT in the Pathophysiology of Cancer.. Front Oncol..

[17] Koppula P, Zhuang L, Gan B (2021). Cystine transporter SLC7A11/xCT in cancer: ferroptosis, nutrient dependency, and cancer therapy.. Protein Cell..

[18] Diao J, Jia Y, Dai E, Liu J, Kang R, Tang D (2024). Ferroptotic therapy in cancer: benefits, side effects, and risks.. Mol Cancer..

[19] Wei MJ, Huang KL, Kang HF, Liu GT, Kuo CY, Tzeng IS (2024). Jing Si Herbal Tea Modulates Macrophage Polarization and Inflammatory Signaling in LPS-Induced Inflammation.. Int J Med Sci..

[20] Kuo CY, Chiu V, Hsieh PC, Huang CY, Huang SJ, Tzeng IS (2020). Chrysophanol attenuates hepatitis B virus X protein-induced hepatic stellate cell fibrosis by regulating endoplasmic reticulum stress and ferroptosis.. J Pharmacol Sci..

[21] Porter AG, Janicke RU (1999). Emerging roles of caspase-3 in apoptosis.. Cell Death Differ..

[22] Zhang W, Liu Y, Liao Y, Zhu C, Zou Z (2024). GPX4, ferroptosis, and diseases.. Biomed Pharmacother..

[23] Lee J, Roh JL (2022). SLC7A11 as a Gateway of Metabolic Perturbation and Ferroptosis Vulnerability in Cancer.. Antioxidants..

[24] Zhu WW, Liu Y, Yu Z, Wang HQ (2025). SLC7A11-mediated cell death mechanism in cancer: a comparative study of disulfidptosis and ferroptosis.. Front Cell Dev Biol..

[25] He C, Li Q, Wu W, Liu K, Li X, Zheng H (2024). Ferroptosis-associated genes and compounds in renal cell carcinoma.. Front Immunol..

[26] He R, Liu Y, Fu W, He X, Liu S, Xiao D (2024). Mechanisms and cross-talk of regulated cell death and their epigenetic modifications in tumor progression.. Mol Cancer..

[27] Li L, Yang L, Yang L, He C, He Y, Chen L (2023). Network pharmacology: a bright guiding light on the way to explore the personalized precise medication of traditional Chinese medicine.. Chin Med..

